# Supplementary material for: Programmed Death 1 and Cytotoxic T-Lymphocyte-Associated Protein 4 Gene Expression in Peripheral Blood Mononuclear Cells Can Serve as Prognostic Biomarkers for Hepatocellular Carcinoma
Source: Cancers (Basel). 2024 Apr 13;16(8):1493. doi: 10.3390/cancers16081493 (PMC11048418; doi:10.3390/cancers16081493)
Supplement: Supplementary file 1 [file cancers-16-01493-s001.zip › Supplementary tables.pdf]

Supporting Information

Table S1. The expression profiles of AST, ALT, and albumin in serum obtained from HCC patients and healthy donors.

	HCC Patients	Healthy donor	P-value
AST [IU/L]	36.85 ± 20.273	21.30 ± 2.908	< 0.001
ALT [IU/L]	24.17 ± 14.100	21.10 ± 7.695	0.509
Albumin [g/dL]	3.743 ± 0.537	4.600 ± 0.240	< 0.001
