# Supplementary figures and images for: Programmed Death 1 and Cytotoxic T-Lymphocyte-Associated Protein 4 Gene Expression in Peripheral Blood Mononuclear Cells Can Serve as Prognostic Biomarkers for Hepatocellular Carcinoma
Source: Cancers (Basel). 2024 Apr 13;16(8):1493. doi: 10.3390/cancers16081493 (PMC11048418; doi:10.3390/cancers16081493)

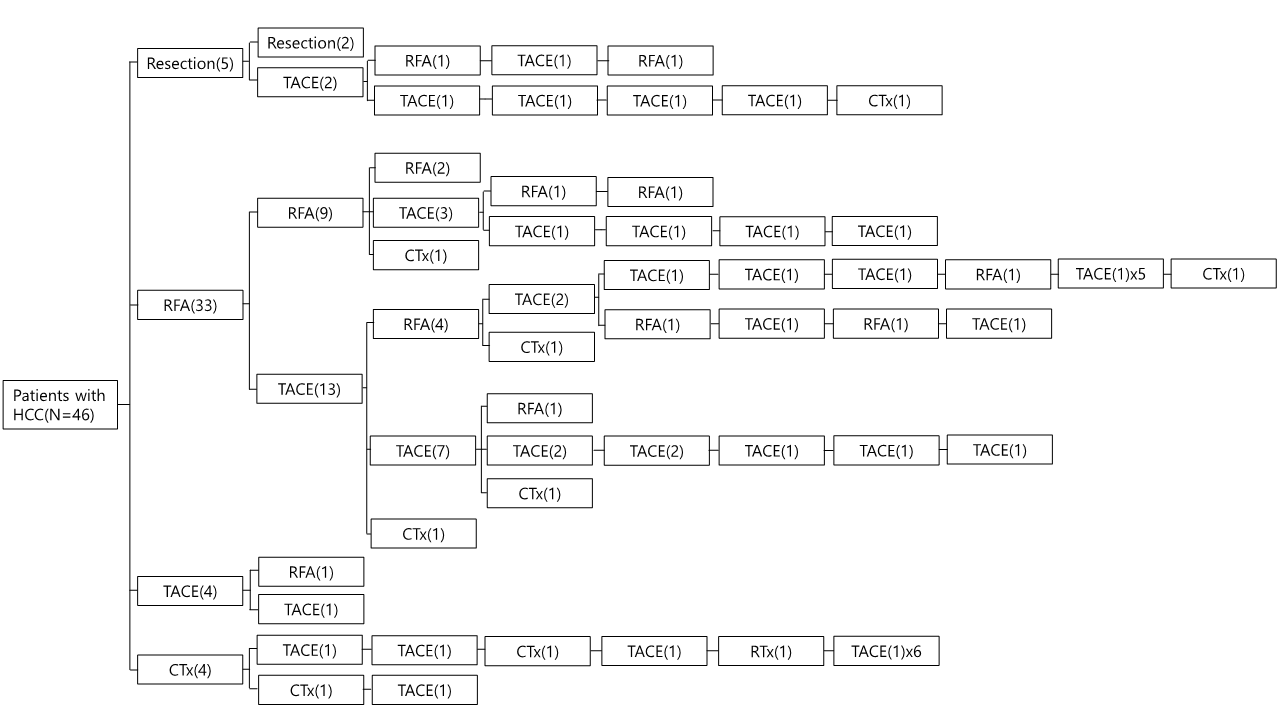

Supplement: Supplementary file 1 [file cancers-16-01493-s001.zip › Figure S1.tif]

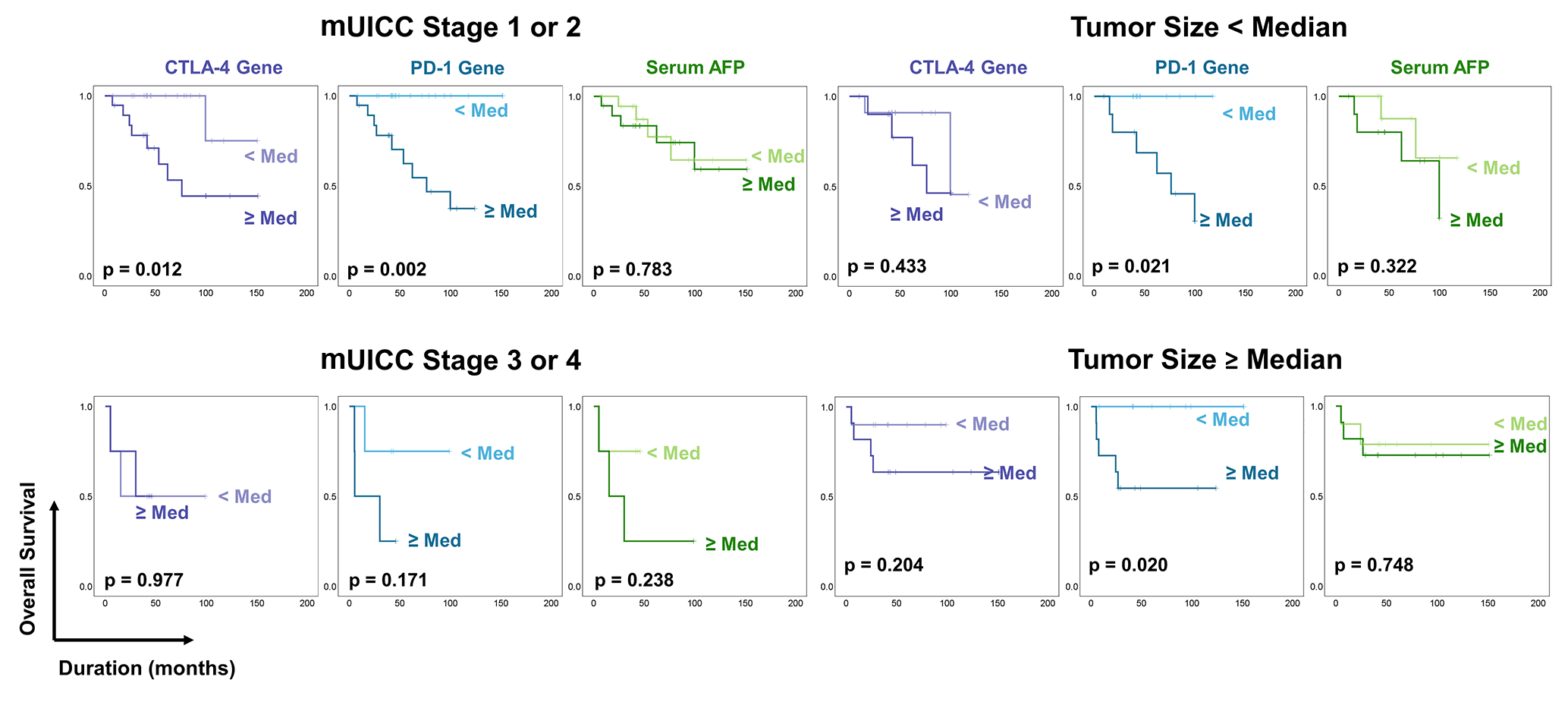

Supplement: Supplementary file 1 [file cancers-16-01493-s001.zip › Figure S5.tif]

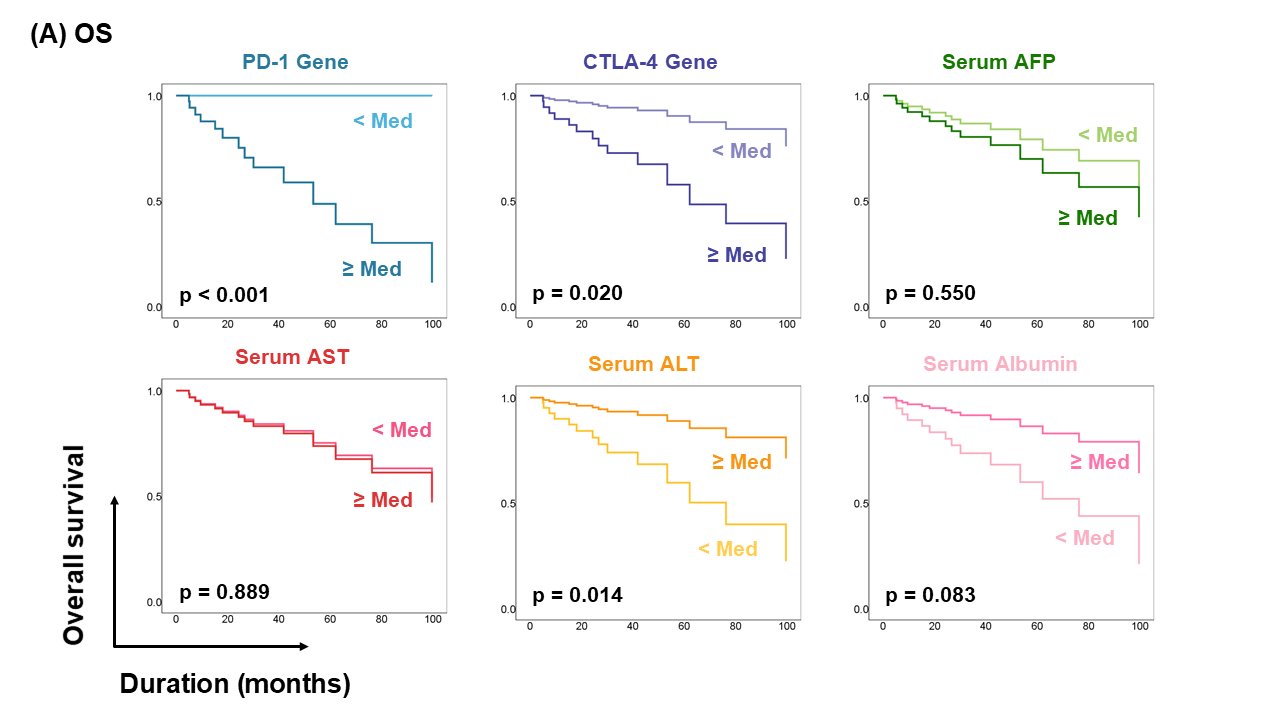

Supplement: Supplementary file 1 [file cancers-16-01493-s001.zip › Figure S6-A.TIF]

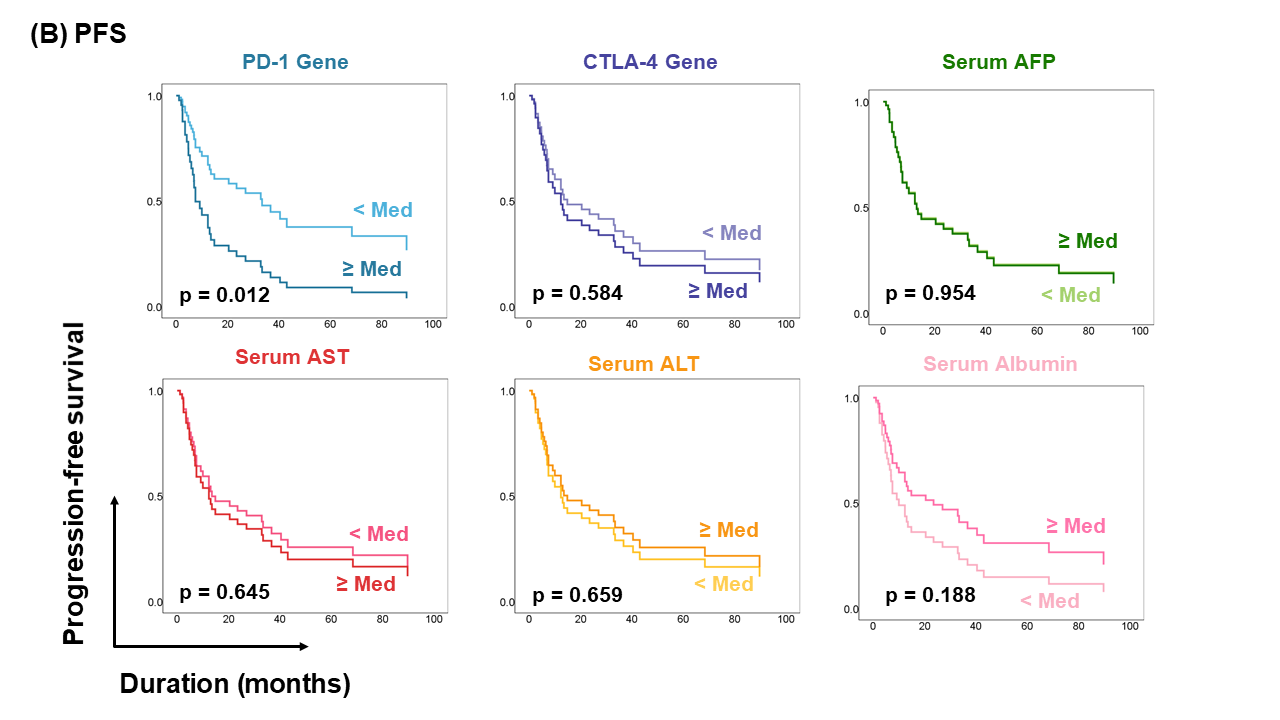

Supplement: Supplementary file 1 [file cancers-16-01493-s001.zip › Figure S6-B.TIF]

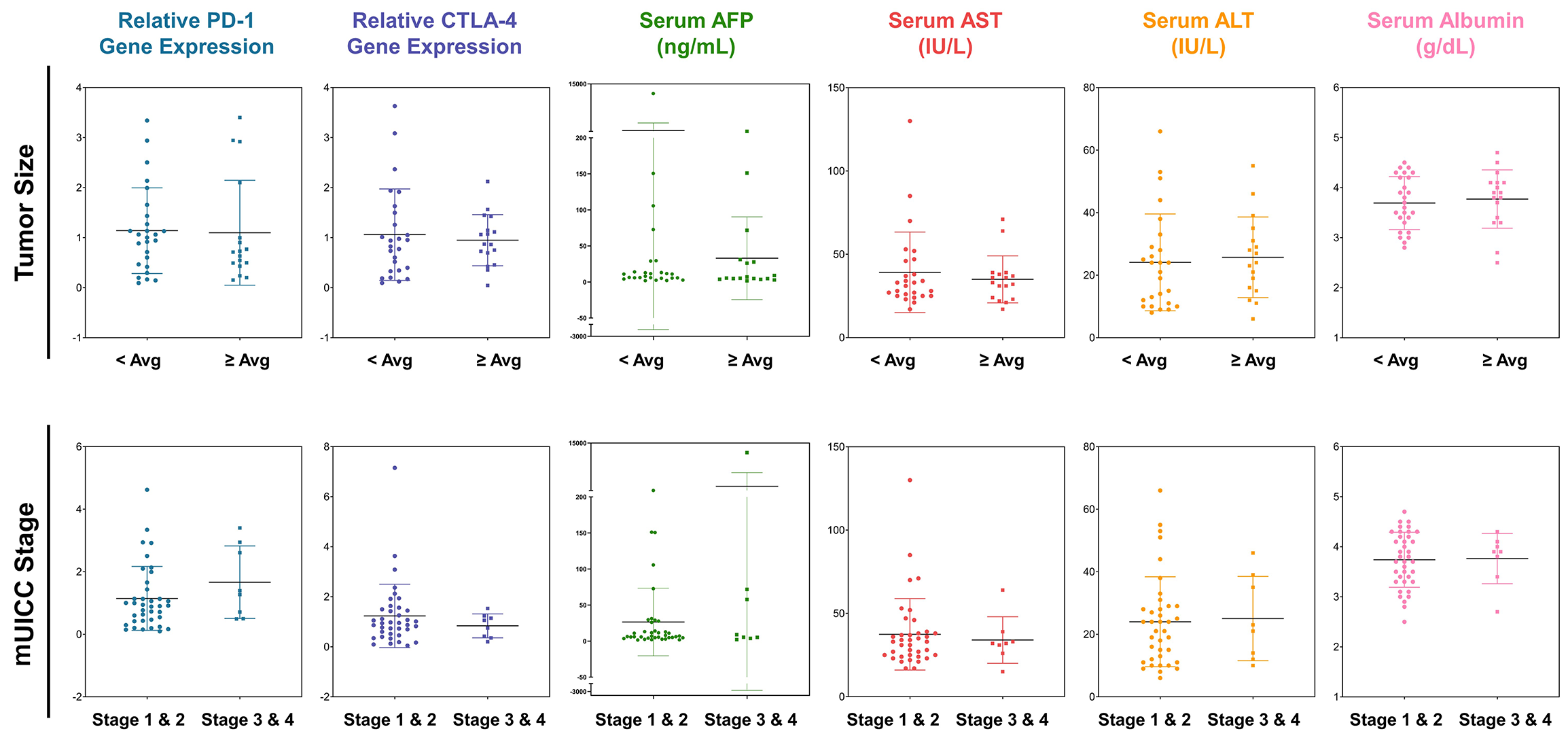

Supplement: Supplementary file 1 [file cancers-16-01493-s001.zip › FigureS2.tif]

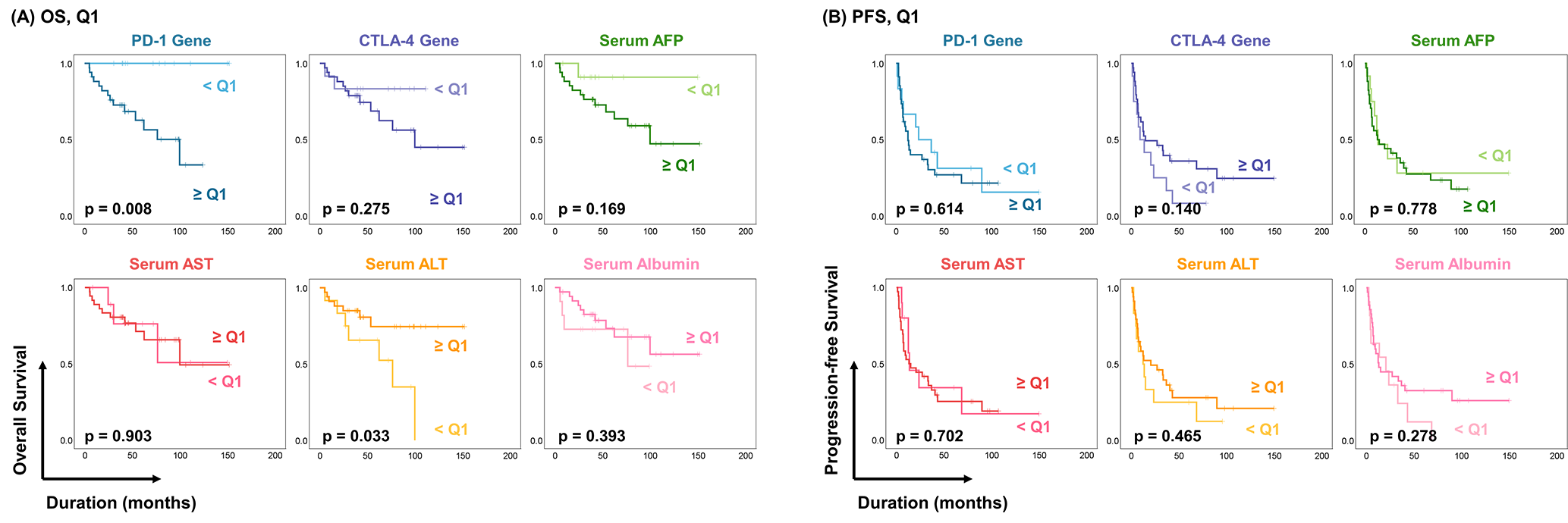

Supplement: Supplementary file 1 [file cancers-16-01493-s001.zip › FigureS3.tif]

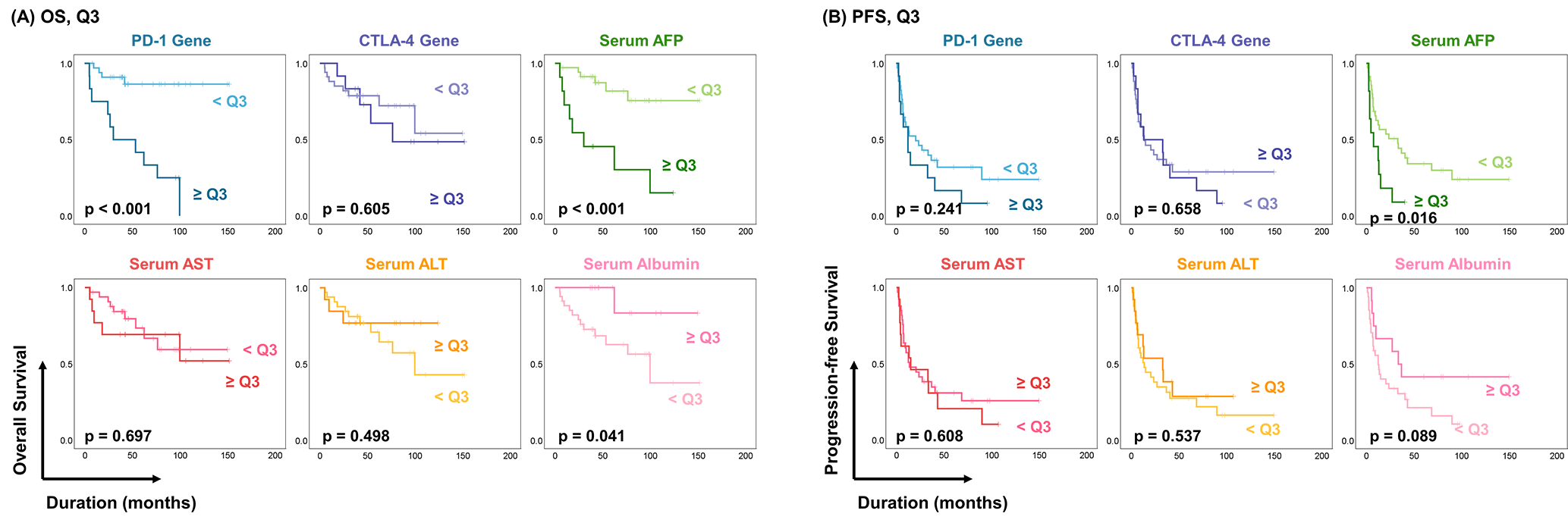

Supplement: Supplementary file 1 [file cancers-16-01493-s001.zip › FigureS4.tif]
